# China’s coal mine methane regulations have not curbed growing emissions

**DOI:** 10.1038/s41467-018-07891-7

**Published:** 2019-01-29

**Authors:** Scot M. Miller, Anna M. Michalak, Robert G. Detmers, Otto P. Hasekamp, Lori M. P. Bruhwiler, Stefan Schwietzke

**Affiliations:** 1 Department of Global Ecology, Carnegie Institution for Science, 260 Panama St., Stanford, CA 94305 USA; 2 SRON, Netherlands Institute for Space Research, 3584 CA Utrecht, Netherlands; 3 Global Monitoring Division, National Oceanic and Atmospheric Administration, 325 Broadway R/GMD 1, Boulder, CO 80305 USA; 4 Cooperative Institute for Research in Environmental Sciences, University of Colorado Boulder, 216 UCB, Boulder, CO 80309 USA; 5 Present Address: Department of Environmental Health and Engineering, Johns Hopkins University, 3400 N. Charles Street, Baltimore, MD 21218 USA; 6 Present Address: Environmental Defense Fund, 2060 Broadway, Boulder, Colorado 80302 USA

## Abstract

Anthropogenic methane emissions from China are likely greater than in any other country in the world. The largest fraction of China’s anthropogenic emissions is attributable to coal mining, but these emissions may be changing; China enacted a suite of regulations for coal mine methane (CMM) drainage and utilization that came into full effect in 2010. Here, we use methane observations from the GOSAT satellite to evaluate recent trends in total anthropogenic and natural emissions from Asia with a particular focus on China. We find that emissions from China rose by 1.1 ± 0.4 Tg CH_4_ yr^−1^ from 2010 to 2015, culminating in total anthropogenic and natural emissions of 61.5 ± 2.7 Tg CH_4_ in 2015. The observed trend is consistent with pre-2010 trends and is largely attributable to coal mining. These results indicate that China’s CMM regulations have had no discernible impact on the continued increase in Chinese methane emissions.

## Abstract (web summary)

Chinese government has implemented regulations to reduce mining-related methane emission since 2010. Here the authors estimated methane emissions in China using GOSAT satellite observation and results reveal a business-as-usual increase in methane emissions since 2010 despite those ambitious targets.

## Introduction

China is the world’s largest producer and consumer of coal (ref. ^1^, Supplementary Fig. 1), and coal accounts for ~72% of the country’s electricity generation (as of 2015, ref. ^2^). This reliance on coal has widely-recognized, adverse impacts on China’s air quality. For example, coal burning contributes 40% of China’s total, population-weighted PM2.5 exposure and 366,000 premature deaths as of 2013^3^.

China’s coal consumption also has an outsized influence on global greenhouse gas (GHG) emissions. China is the world’s largest anthropogenic emitter of methane gas (CH_4_) according to some estimates^4^, and the coal sector contributes the highest fraction of the country’s anthropogenic CH_4_ emissions (~33%)^4^. CH_4_ accumulates in coal seams during the process of coalification—when organic material is slowly converted into coal over geological time scales^5^; the majority of coal-related CH_4_ emissions occur when the coal is mined and this trapped CH_4_ gas is released to the atmosphere (e.g., refs. ^6,7^).

Coal production has been increasing in China, at least until 2015^1,8^. Coal production increased 2.5-fold between 2000 and 2010—from 1384 to 3428 million metric tons^1^. Existing emissions inventories, however, provide divergent estimates on how this increase affected China’s CH_4_ emissions. For example, the US Environmental Protection Agency (EPA) estimates a trend of 0.33 Tg CH_4_ yr^−1^ while the Emission Database for Global Atmospheric Research inventory (EDGAR, v4.3) puts the trend at 1.5 Tg CH_4_ yr^−1^ (mean trend for 2005–2010)^4,9^. Estimates based on observations of atmospheric CH_4_, on the other hand, are relatively consistent in reporting a trend of 1.0 to 1.2 Tg CH_4_ yr^−1^ between 2000 to 2010^10–12^, with only one study estimating a trend as high as 2.0 Tg CH_4_ during a subset of these years^12^. This annual increase is larger than total annual anthropogenic CH_4_ emissions from countries like Greece or the Netherlands^4^.

The national government, however, has set ambitious benchmarks for the utilization of CH_4_ produced during the coal mining process (referred to as coal mine methane or CMM). China’s twelfth Five Year Plan specifies that total CMM utilization should have been 8.4 billion cubic meters or 5.6 Tg of CH_4_ by 2015. Targets for 2020 are even more ambitious; CMM recovery should be 13.2 Tg CH_4_ (20 billion cubic meters) by that date, and a large majority of this production should be utilized, not flared or vented^13^. To reach these CMM goals, beginning in 2006, the State Council required that all coal companies drain mines of CH_4_ prior to coal production and declared that coal mines cannot legally operate without CMM drainage systems^14^.

Subsequently, the national government enacted a policy requiring that mines either utilize or flare all drained CH_4_, a policy that became effective for all coal mines beginning in 2010^14^. These regulatory requirements have been paired with financial incentives. Mine operators receive a monetary subsidy for all utilized CMM and receive a mandatory price premium for the resulting electricity that is sold to the grid. Grid companies are further required to prioritize electricity produced from CMM^14,15^. The utilized CH_4_ is also exempt from licensing fees and royalties^16^. These policies, however, have a notable caveat. Mine operators are exempt from flaring and utilization requirements if the drained gas has a CH_4_ content <30%. This is because CH_4_ at concentrations between 5–16% is explosive due to the high O_2_ to CH_4_ ratio and is therefore dangerous to transport or flare^17^.

Existing evidence indicates that these targets, regulations, and incentives for CMM flaring and/or utilization are ambitious. CMM utilization jumped from 0.6 and 2.3 Tg CH_4_ between 2005 and 2012 (0.9–3.5 billion cubic meters respectively)^18^, but this is well below the 2015 target.

In the present study, we estimate CH_4_ emissions across temperate and tropical Asia for 2010–2015, specifically focusing on China to explore the extent to which environmental regulations and structural changes have impacted CH_4_ emissions from the country. We do so using 6.5 years of CH_4_ observations from the GOSAT satellite (e.g., ref. ^19^) paired with a global atmospheric model and an atmospheric inversion to estimate emissions.

## Results and Discussion

### Global trends in GOSAT observations

**Fig. 1:**
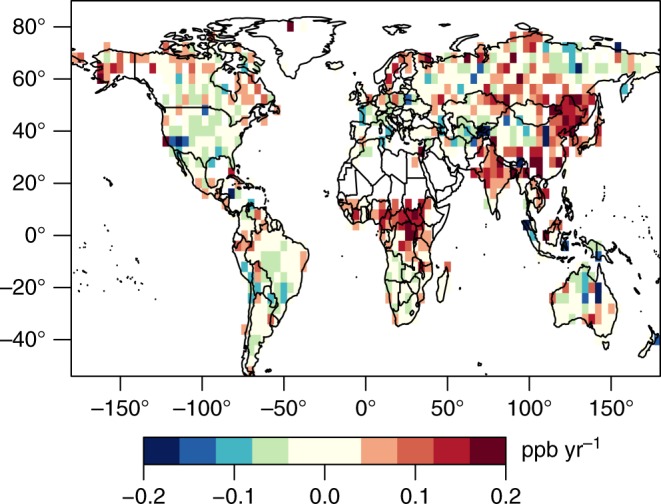
Trend in nadir GOSAT observations. The figure displays the trend between September 2009 and September 2015 minus the trend in the NOAA global marine observations. The GOSAT observations are averaged into 2.0° × 2.5° latitude–longitude boxes before fitting the trend, and the figure only displays boxes with more than 250 total observations. Red colors indicate that the GOSAT observations are increasing faster than the NOAA global marine average while green and blue colors indicate an increase slower than the NOAA average. China, India, tropical Africa, and tropical Asia show increases that are faster than the global average

Figure 1 displays the trend in GOSAT observations between September 2009 and September 2015 for aggregate 2.0° × 2.5° latitude–longitude grid boxes. The trend in Fig. 1 is relative to the trend in the National Oceanic and Atmospheric Administration (NOAA) globally averaged marine monthly mean data^20^. These trends are also independent of changes in global average hydroxyl radical mixing ratios because they are relative to a global background trend.

The trend in atmospheric CH_4_ mixing ratios in tropical Africa, sub-tropical Asia, and temperate Asia is large relative to the global mean trend (Fig. 1). Atmospheric CH_4_ has been increasing globally since 2007 (e.g., refs. ^21,22^). This trend in sub-tropical Asia and tropical Africa is consistent with existing studies that show tropical regions have been driving these recent global CH_4_ increases (e.g., refs. ^21,22^), and variability in these tropical fluxes is not well captured in existing bottom-up models (e.g., ref. ^23^).

By contrast, other regions of the globe do not exhibit such clear trends relative to the global mean. For example, the trend in many areas of the US is likely nominal relative to the global marine trend (Fig. 1). Additional analysis of US CH_4_ trends are beyond the scope of the present study. Additionally, there is no clear pattern in Fig. 1 across the Amazon or adjacent regions. Large fires in the Amazon in 2010 emitted a pulse of CH_4_ to the atmosphere, making it difficult to fit a simple multi-year trend for the region (e.g., ref. ^24^). Further, note that trends at high latitudes are highly uncertain due to data sparsity (e.g., ref. ^25^), and we do not discuss these regions in detail here.

### Trends in emissions from China and Asia

Changes in atmospheric CH_4_ mixing ratios over a particular region can be due to CH_4_ emissions in that or any upwind regions, and an inverse modeling framework can be used to attribute patterns in atmospheric CH_4_ to patterns in surface emissions.

**Fig. 2:**
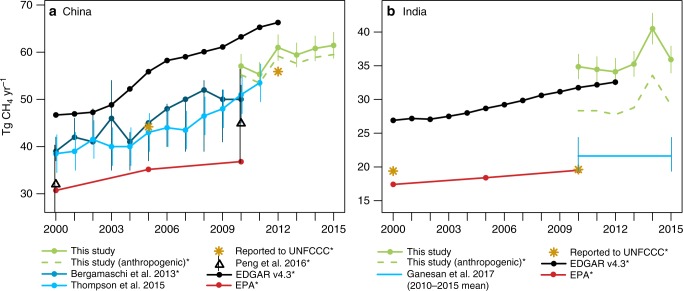
Methane emissions estimates for China and India. **a** CH_4_ emissions estimates for China from this study, Bergamaschi et al.^10^, Thompson et al.^12^, UNFCCC^35^, Peng et al.^6,^ the EDGAR v4.3 inventory^4^, and the US EPA^9^; and **b** for India from this study, Ganesan et al. ^28^, UNFCCC^53^, and US EPA^9^. We find a trend in emissions from both countries for 2010–2015, though the trend for India is uncertain. Note that uncertainty estimates for this study are 95% confidence intervals, and uncertainty bounds for Bergamaschi et al.^10^ reflect the range of different inversions that use different datasets (e.g., in situ, satellite). Estimates marked with an asterisk are for anthropogenic emissions only. Furthermore, the dashed green line represents the posterior emissions estimate after subtracting the wetland emissions model, biomass burning inventory (GFED), and termite emissions

**Fig. 3:**
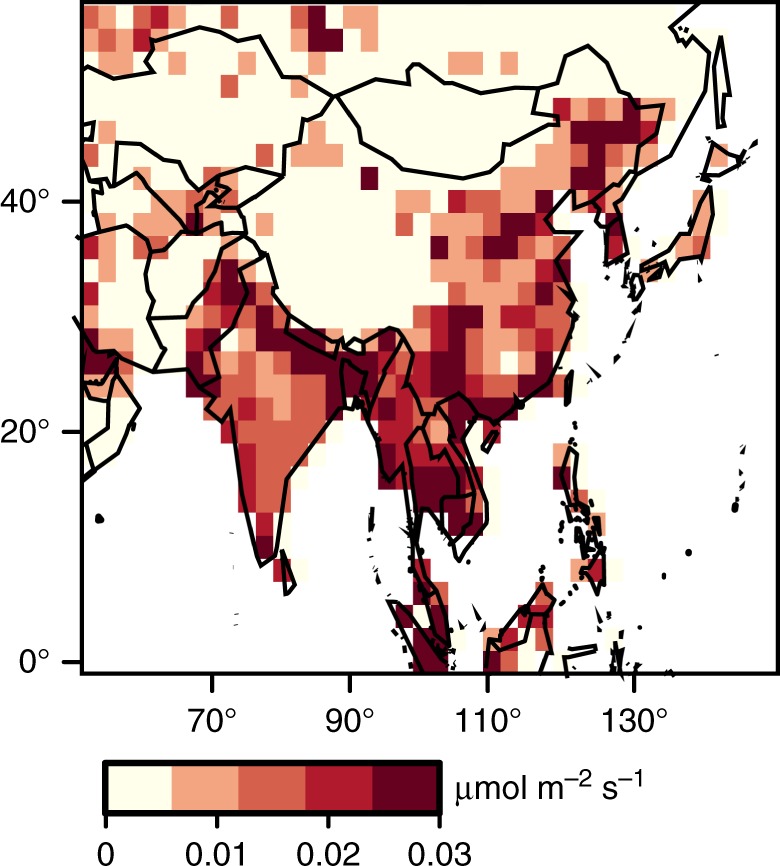
Map of CH_4_ emissions estimates. Total CH_4_ emissions (anthropogenic plus natural) estimated using GOSAT observations and the inverse model (2010–2015 mean). CH_4_ emissions from China are highest in provinces with large coal production and coal formations that contain high amounts of CH_4_ (e.g., Shanxi, Guizhou, and Anhui; refer to Supplementary Fig. 1). Note that the inverse modeling emissions estimate is highly uncertain for any individual grid box, but those uncertainties decrease at increasing spatial scales (Supplementary Fig. 2)

We incorporate atmospheric CH_4_ observations into an inverse model and find an increasing trend in CH_4_ emissions across much of Asia, including in China and India (Figs. 2 and 3, Supplementary Figs. 2 and 3). The trend in total emissions from China is 1.1 ± 0.4 Tg CH_4_ yr^−1^ (*p* = 0.058). Globally, CH_4_ emissions have been increasing at a rate of ~5 to 8 Tg CH_4_ yr^−1^ since 2007 (e.g., refs. ^10,20^). The emissions increase from China accounts for ~11–24% of this total global trend (95% confidence interval). The estimated trend in Indian emissions is less certain, on the other hand, at 0.7 ± 0.5 Tg CH_4_ yr^−1^ (*p* = 0.25).

These results show that the reported trend in China’s CH_4_ emissions prior to 2010 has continued in subsequent years, in spite of regulations aimed at substantially reducing coal mining emissions. Top–down atmospheric studies generally indicate an annual trend of 1.0–1.2 Tg CH_4_ for the 2000s (e.g., refs. ^10–12^), and we find that a trend of the same magnitude has continued past 2010. The results of this and earlier studies collectively indicate that China’s annual CH_4_ emissions increased by ~50% between 2000 and 2015 (~20 Tg CH_4_), an increase comparable to total annual anthropogenic CH_4_ emissions from countries like Russia and Brazil^4^. By comparison, this increase is more modest than reported in earlier versions of EDGAR (3.3 Tg CH_4_ yr^−1^ mean trend in EDGAR v4.2 for 2000–2010) but is similar to the newer version of EDGAR (1.6 Tg CH_4_ yr^−1^ average trend in EDGAR v4.3 for 2000–2012).

**Fig. 4:**
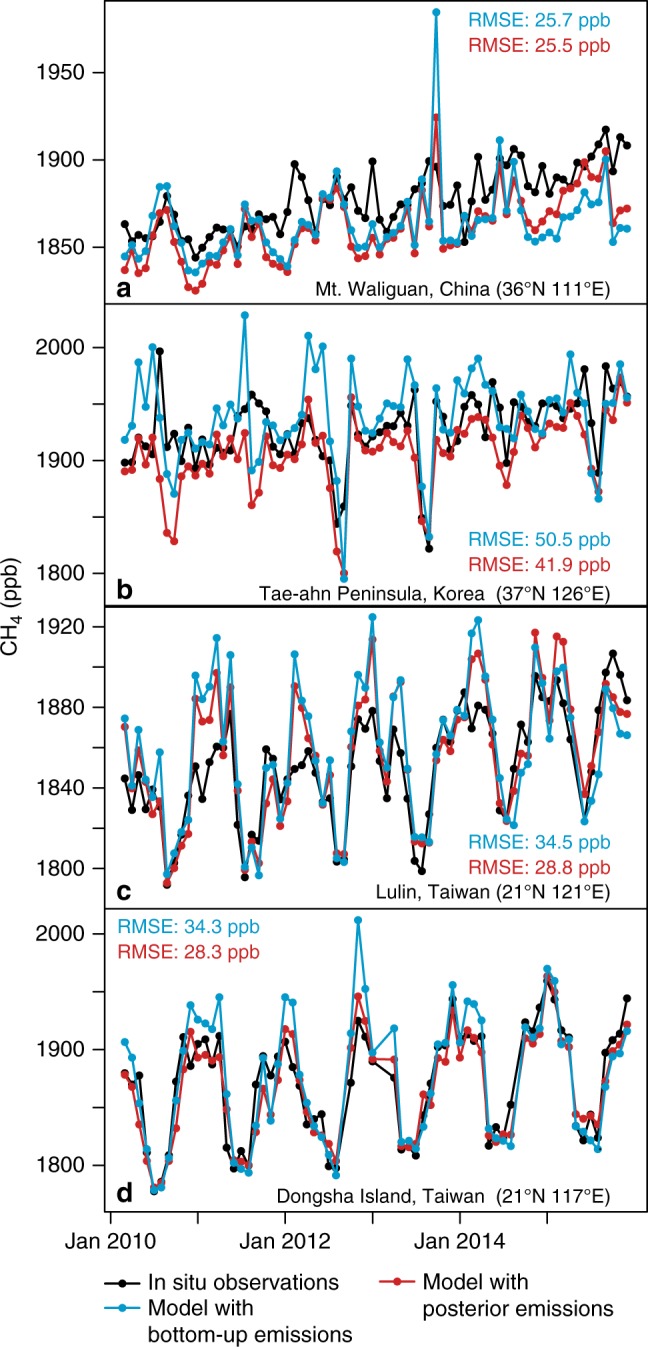
Observed and modeled CH_4_ in situ mixing ratios. The sites shown are in situ monitoring stations from the NOAA Global Greenhouse Gas Reference Network^20^. Modeled CH_4_ using the posterior emissions shows a lower root mean squared error (RMSE) at all sites. Note that all panels display monthly model and data means, and the blue modeled time series in this plot use EDGAR v4.2, the same inventory version used in the inverse model

The estimated emissions for India and China are also in good agreement with available in situ CH_4_ observations across Asia. Modeled total column CH_4_ using the estimated emissions have a smaller bias and correlate better with in situ observations relative to the prior emissions. Figure 4 displays model–data comparisons at four in situ sites—in China, Korea, and Japan. Two of these sites are mountain-top sites (panels a and c) while two are marine sites (panels b and d). None of these sites are included in the inverse model, providing an independent check on the emissions estimated using GOSAT observations.

Note that the total emissions estimate for India is in the mid-range of existing, global inverse modeling studies^26,27^. These earlier studies compare results from multiple global inverse models and report multi-model averages of 33 and 39 Tg yr^−1^, respectively (for 2000–2009 and 2003–2012, respectively). The emissions reported here (36 ± 2.5 Tg yr^−1^) are consistent with those studies. By contrast, a recent, regional inverse modeling study of India is an outlier compared to these studies at 22 Tg yr^−1^ (Fig. 2, 2010–2015 mean)^28^.

### Contribution of various source sectors

**Fig. 5:**
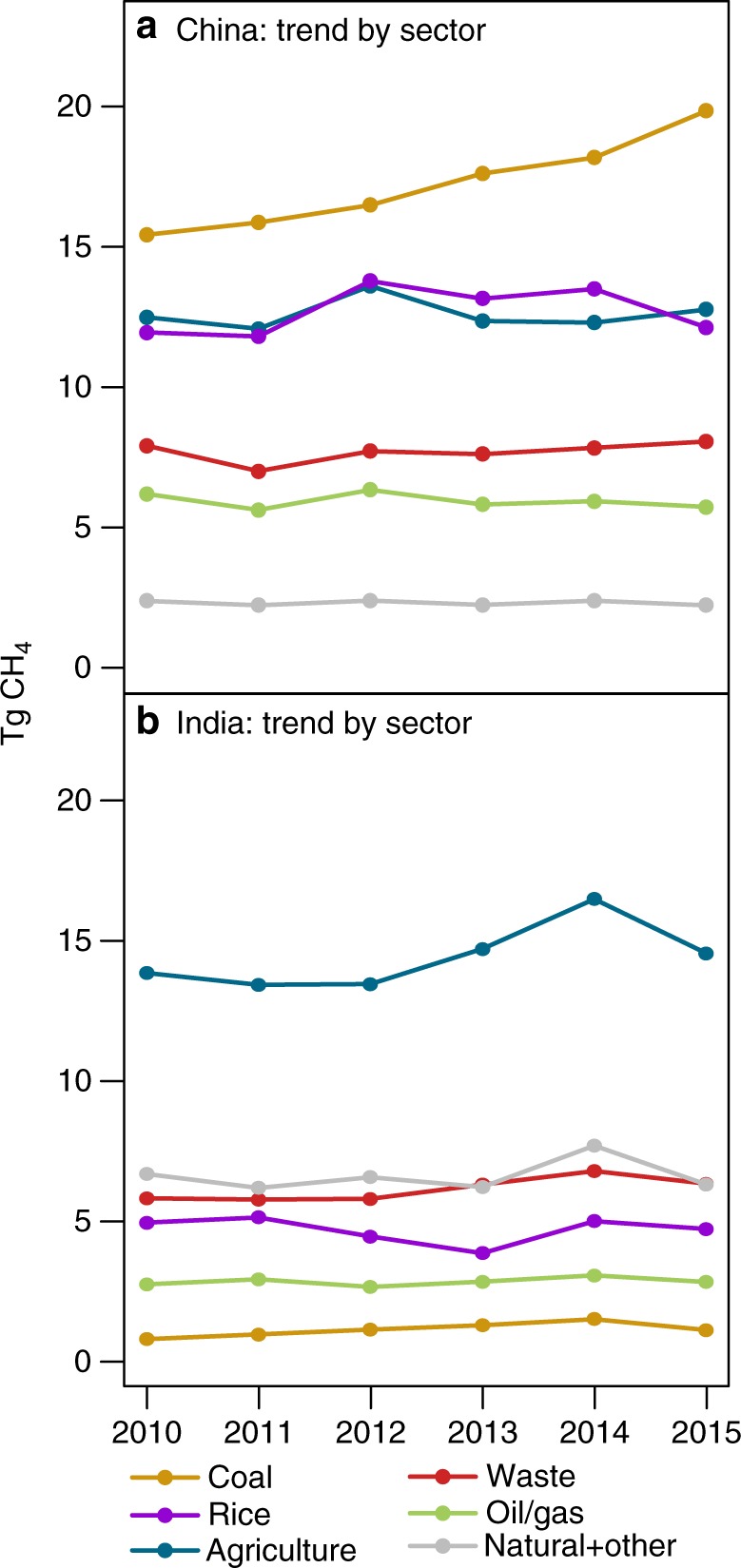
Emissions trends by sector. **a**, **b** show the estimated emissions trend by sector for China and India, respectively. The coal sector appears to be driving the trend in China. No clear trends are obvious for India. Note that all trends in this figure are driven by the GOSAT observations, not by the EDGAR anthropogenic emissions inventory used in the inverse model; the inventory estimate is constant with time. The EDGAR inventory does not include uncertainty estimates for the sector-specific breakdown of emissions, and uncertainty estimates are therefore not included here. Figure 3 and Supplementary Fig. 2 present uncertainties for the total emissions

We find a clear trend in CH_4_ emissions from China’s coal sector while other source sectors do not show a corresponding trend (Fig. 5). By contrast, no source sector in India shows an obvious trend; the trend in total emissions from India is uncertain, and it is not clear what could be driving that trend, if one exists. This attribution is based upon the emissions estimate from the inverse model and the spatial distribution of different source sectors within the EDGAR emissions inventory. Specifically, we attribute emissions within each individual model grid box based upon the relative fraction of emissions that are due to each sector within that grid box in the EDGAR emissions inventory. Refer to the Methods for additional detail.

Additional lines of evidence also indicate that coal is likely driving the overall trend in China’s emissions. Coal production in China increased between 2010 and 2015 (from 3400 to 4000 million metric tons^1^) whereas ruminant populations and rice production have remained flat or grown only slightly. For example, milled rice production grew from 137,000 thousand metric tons in 2010/2011 to 140,850 thousand metric tons in 2016/2017^29^. Beef production increased by only 8% between 2011 and 2016, and China’s dairy cattle inventory declined due to both decreasing dairy demand and increasing dairy and beef imports^30,31^.

### Implications for coal mine methane

Overall, results indicate that CH_4_ emissions from China have been increasing since 2010 and that this increase has not slowed. Existing bottom–up and top–down studies disagree on the magnitude and trend in CH_4_ emissions from China (e.g., refs. ^26,27^), and the present study sheds additional light on these emissions. We find that, although China has set ambitious benchmarks, regulations, and incentives for CMM drainage and utilization since the mid-2000s, emissions continue to increase following a business-as-usual scenario. This increase in emissions is most likely driven by the coal sector, implying that China’s ambitious coal CH_4_ actions have not produced a detectable change in the rate of increase in CH_4_ emissions.

Existing studies from the US EPA and the International Energy Agency (IEA) have identified three broad barriers that China would need to overcome to meet its CMM targets (e.g., refs. ^14,15,32^). One or more of these barriers has presumably hampered China’s progress, and these studies help place the results presented here within a broader policy context.

First, insufficient infrastructure makes it difficult to bring CMM to market, and the US EPA cites this challenge as a potential barrier to achieving China’s CMM goals^15,32^. Most coal mines are located in remote mountainous areas, areas that are poorly connected to cities or natural gas infrastructure (ref. ^32^, ch. 7). Furthermore, the US EPA describes China’s gas market as “underdeveloped”, and only 22% of China’s non-rural population had access to natural gas as of 2010^15^.

Second, inadequate technology likely presents an obstacle. Most coal mines in China are deep, and the coal seams are highly impermeable, unlike many mines in the US and Australia. The CMM drainage technology often used in China is poorly suited for these conditions^14,32^. As a result, the resulting CMM is often of poor quality (i.e., low CH_4_ content), according to US EPA^15^. In addition, the IEA explains that operators of small and medium mines often lack the technical expertize to utilize the CH_4_ for heating or electricity production^14^.

Third, inadequate or poorly-designed policies may stand in the way of reaching CMM utilization targets. US EPA explains that existing regulations and incentives have not been fully realized, and some may have backfired^15^. Utility companies often resist accepting electricity generated from CMM, in spite of policies that require utilities to give priority to this electricity. According to US EPA^15^, “The incentive program for CMM power plant utilization proved particularly difficult to implement due to resistance from power grid companies uneager to manage the complexities of dispatch of the fluctuating output of small CMM plants, and lacking a policy mechanism to pass the premiums through to consumers.” CMM utilization requirements may have also backfired. Government policy requires that all mines utilize drained gas with greater than 30% CH_4_ content. EPA has anecdotal evidence that mine operators may be diluting drained gas to circumvent the requirement^15^. These actions not only render CMM unusable but also unsafe. In addition, the IEA points out that most local and provincial governments have limited power to enforce CMM regulations, limiting overall enforcement action^14^.

**Fig. 6:**
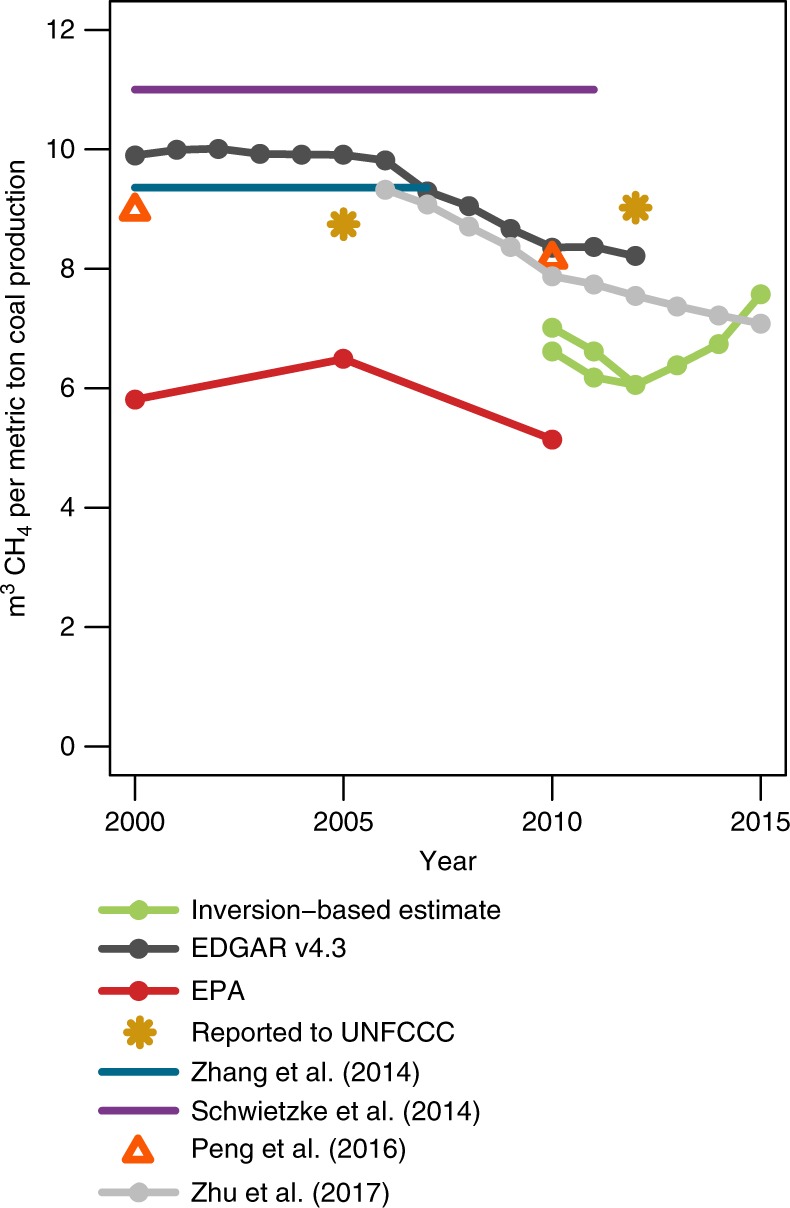
Coal CH_4_ emissions factors. Emissions factors implied by this study, EDGAR v4.3^4^, US EPA^9^, UNFCCC^35^, Zhang et al.^54,^ Schwietzke et al.^55,^ Peng et al.^6,^ and Zhu et al.^7^. The factors implied by this study are in the lower mid-range of existing estimates. In some cases, we divide total emissions by coal production numbers to derive emissions factors for this figure. For example, we divide the EDGAR emissions estimate by EIA coal production estimates^1^, and we divide Peng et al.^6^ and China’s UNFCCC report by production numbers from China’s Statistical Yearbook^8^, the production numbers used in those estimates. The two green lines in this figure are the inversion estimate divided by production numbers from EIA and China’s Statistical Yearbook, respectively. Note that Schwietzke et al.^55^ and Zhang et al.^54^ use time-invariant emissions factors, and these studies are therefore represented with solid lines

Existing inventories diverge on how China’s coal CH_4_ emissions have changed since 2010 (e.g., refs. ^4,9^). Emissions factors (i.e., leak rates) provide a convenient means to compare these inventory emissions and estimated trends. Emissions factors in existing inventories range from ~5 to 11 m^3^ of CH_4_ per metric ton of coal mined (weighted national average) (Fig. 6). We find emissions factors of ~6–7 m^3^ of CH_4_ per metric ton, depending upon the year, by dividing coal emissions estimates presented here (Fig. 5) by China’s total coal production^1,8^. Note that we divide China’s official emissions estimate by its own coal production numbers while we divide the EDGAR emissions inventory by coal production numbers from the Energy Information Administration (EIA)^1,8^. These two coal production estimates contain similar trends but differ by up to ~5–10% in some years.

These emissions factors are similar to those in several existing inventories, but the trend is not. The emissions factors in many inventories have been declining with time and are forecasted to continue declining in future years (Fig. 6). These declines are often due to assumptions about improved energy technology and the forecasted effects of environmental regulations (e.g., ref. ^7^). By contrast, the emissions factors implied by this study show a slight upward trend from 2011 onward. This upward trend could be real, it could be due to uncertainties or errors in the inverse model and the associated source attribution, or it could point to inaccuracies in China’s coal production statistics. The EIA’s coal production numbers for China are uncertain, and there have recently been large discrepancies and an upward revision in China’s coal production and consumption statistics^33,34^. If the EIA were to underestimate China’s production trend, it could alias into the emissions factors and create a spurious upward trend. Note that the emissions factors from China’s National Bureau of Statistics also show a slight upward trend between 2005 and 2012. China submitted an explanation of its provincial coal emissions factors in 2012 along with its emissions estimate for 2005^35^. We suspect that China used the same emissions factors in its 2012 emissions update, and changes in the emissions factor between 2005 and 2012 in Fig. 6 more likely reflect a shift in coal production to provinces with higher coal CH_4_ content.

Overall, we find that China’s CH_4_ emissions have continued to increase unabated since 2010, likely driven by increasing coal production (Fig. 5). Specifically, estimated CH_4_ emissions increases are highest in regions where the EDGAR inventory indicates a predominance of coal mining emissions relative to other source types (Supplementary Figs. 1 and 3, refer to Methods). Furthermore, coal production in China continues to increase while cattle counts and rice production have remained relatively flat during the study period. These results imply that China’s regulations and initiatives have not produced a detectable flattening or decline in CH_4_ emissions. China has an opportunity to mitigate substantial CH_4_ emissions through CMM drainage and utilization (or flaring), and the national government in China has taken steps to require more environmentally friendly practices. Observations from GOSAT indicate a business-as-usual emissions scenario up to 2015, and it is therefore unlikely that China has met its ambitious regulatory goals.

## Methods

### GOSAT observations

The Greenhouse Gases Observing Satellite (GOSAT) was launched in January of 2009 by the Japan Aerospace Exploration Agency (JAXA). The satellite flies in a sun-synchronous polar orbit, passing each location at ~13:00 local time (e.g., ref. ^19^). GOSAT is sensitive to CH_4_ mixing ratios throughout the troposphere, and this sensitivity slowly declines in the stratosphere at increasing altitudes (e.g., ref. ^36^).

All of the analyses conducted in this paper use CH_4_ observations generated using the RemoteC v2.3.8 proxy retrieval (e.g., ref. ^37^). The v2.3.8 retrieval and its evaluation are described in detail in Hasekamp et al.^38^ and Supplementary Note 1. A study comparing this retrieval against TCCON observations^39^ indicates that the two types of observation are in good agreement^37^. Furthermore, inverse modeling estimates based on data from this retrieval are consistent with estimates based on data from the global network of in situ CH_4_ observations^40^. We use high gain nadir observations and exclude glint observations along with any observations that have a negative quality control flag. The resulting dataset has an average of 2.2 × 10^5^ observations per year during the study period (e.g., Supplementary Fig. 4).

The GOSAT observations and modeled total column mixing ratios show a latitude-dependent difference or bias (Supplementary Note 2). Two previous modeling studies found a comparable bias^41,42^. This bias could be due to the GOSAT observations or the atmospheric model. Existing studies have not pinpointed a cause but speculate that the bias may be due to model–GOSAT differences in the stratosphere. We apply a latitude-dependent correction to the GOSAT observations to remove this bias. We use the same procedure as a recent study^42^ and describe the correction in greater detail in Supplementary Note 2 and in Supplementary Figs. 5 and 6. This correction ranges from approximately −10 to −15 ppb across East Asia, depending upon the latitude.

### Inverse model

We use the GEOS-Chem (Goddard Earth Observing System—Chemistry) model to simulate atmospheric transport as part of the solution of the inverse problem (e.g., refs. ^42,43^). Supplementary Note 3 describes these simulations in greater detail.

We use a combination of inventory estimates within the inverse model. The EDGAR emissions inventory (Emission Database for Global Atmospheric Research, version 4.2) serves as the anthropogenic emissions estimate^44^, an online wetland model provides daily wetland emissions^43^, and daily biomass burning emissions are from the Global Fire Emissions Database version 4^45,46^. The anthropogenic inventory used in the inversion (EDGAR)^4^ is time-invariant for the setup here, such that any estimated emissions trends are solely due to the GOSAT observations.

The inverse model will scale the total emissions (anthropogenic plus natural) in each model grid box (2.0° × 2.5° latitude-longitude) within Asia and will estimate a different emissions scaling factor for each TransCom region (e.g., ref. ^47^). This setup provides computational savings outside the region of interest, and the global scope of the inversion also ensures that the air masses entering the domain of interest are consistent with global observations. We estimate a different set of scaling factors for each 6-month block of the 6.5 year study time period.

The inverse model employed here is Bayesian. It accounts for errors in the model and measurements (i.e., model-data mismatch errors) and errors in the prior emissions estimate. We also account for spatial and temporal covariances in both the model-data mismatch and prior errors by including off-diagonal elements in the associated error covariance matrices. This setup ensures more realistic uncertainties on the estimated fluxes. Supplementary Notes 4–7 describe this setup and the uncertainty calculations in greater detail. In addition, Supplementary Note 8 discusses the inverse model in context of CH_4_ isotopes.

Supplementary Note 9 provides further discussion of how global changes in the hydroxyl radical (OH) could impact the inverse modeling results. Specifically, it is unlikely that the emissions trends estimated in this study are due to a trend in OH. Some studies argue that OH may be changing^48,49^ while other studies find no evidence for recent changes in OH^50,51^. Even if OH levels were changing, the resulting trend in atmospheric total column CH_4_ would likely be small relative to the regional trends across China in Fig. 1.

### Sector attribution

We also investigate which emissions sectors are most likely to be responsible for emissions from China and India, and more importantly, which sectors may be driving any emissions trends (Supplementary Note 10). Within each grid box, we attribute the same fraction of emissions to each source type as in the corresponding grid box of EDGAR (e.g., refs. ^42,52^). This approach does not assume that EDGAR estimates correct emissions totals for each grid box. However, it assumes that EDGAR attributes the correct fraction of emissions by sector within each grid box. Two different errors could bias this fraction and create uncertainty in the source attribution in this study: inaccuracies in the spatial distribution of each source sector and an over or underestimation of an individual source sector. The former problem (incorrect spatial distribution) appears unlikely; the spatial distribution of coal mining in EDGAR is likely accurate for China because the locations and characteristics of coal mining regions in China are well-known. We investigate the latter issue (over- or underestimate a source type) in detail in Supplementary Note 10 and Supplementary Fig. 7. We conclude that this issue does add some uncertainty to the source attribution, but the overall conclusions are robust.

## Supplementary information

Supplementary Information

Peer Review File

## Data Availability

All of the satellite and in situ CH_4_ data used in this paper are publicly available online. The RemoTeC CH_4_ proxy retrievals (v2.3.8) are available online at ftp://ftp.sron.nl/pub/pub/RemoTeC/C3S/CH4_GOS_SRPR/V2.3.8/. Furthermore, in situ data are archived on the NOAA Global Greenhouse Gas Reference Network: https://www.esrl.noaa.gov/gmd/ccgg/ggrn.php. Other data products used in the study like the EDGAR emissions inventory and Global Fire Emissions Database are available for download at http://edgar.jrc.ec.europa.eu/ and https://www.globalfiredata.org/.

## References

[1] US Energy Information Administration. US Energy Information Administration International energy statistics. *e**ia Beta* https://www.eia.gov/beta/international/data/browser/. Last access: 19 Mar 2018 (2017).

[2] US Energy Information Administration. International Energy Outlook. *eia* https://www.eia.gov/outlooks/aeo/data/browser/#/?id=15-IEO2017®ion=4-12&cases=Reference&start=2010&end=2014&f=A&linechart=Reference-d021916a.2-15-IEO2016.4-12&map=&sourcekey=0. Last access: 19 Mar 2018 (2017).

[3] Health Effects Institute GBD MAPS Working Group. Burden of disease attributable to coal-burning and other major sources of air pollution in China, (Boston, MA). *HEI* https://www.healtheffects.org/publication/burden-disease-attributable-coal-burning-and-other-air-pollution-sources-china. Last access: 19 Mar 2018 (2016).

[4] Janssens-Maenhout G (2017). EDGAR v4.3.2 global atlas of the three major greenhouse gas emissions for the period 1970–2012. Earth Syst. Sci. Data.

[5] Thomas L (2002). Coal Geology.

[6] Peng S (2014). Inventory of anthropogenic methane emissions in mainland China from 1980 to 2010. Atmos. Chem. Phys..

[7] Zhu T, Bian W, Zhang S, Di P, Nie B (2017). An improved approach to estimate methane emissions from coal mining in China. Environ. Sci. Technol..

[8] National Bureau of Statistics of China. *China Statistical Yearbook 2017*. Ch. 9 (China Statistics Press, Beijing, China, 2017).

[9] US Environmental Protection Agency Office of Atmospheric Programs. *Global anthropogenic non-CO*_*2*_*greenhouse gas emissions: 1990–2030*, Technical Report EPA 430-S-12-002. https://www.epa.gov/global-mitigation-non-co2-greenhouse-gases/global-anthropogenic-non-co2-greenhouse-gas-emissions. Last access: 19 Mar 2018 (EPA, Washington, DC, 2012).

[10] Bergamaschi P (2013). Atmospheric CH_4_ in the first decade of the 21st century: inverse modeling analysis using SCIAMACHY satellite retrievals and NOAA surface measurements. J. Geophys. Res. Atmos..

[11] Bruhwiler L (2014). Carbontracker-CH_4_: an assimilation system for estimating emissions of atmospheric methane. Atmos. Chem. Phys..

[12] Thompson RL (2015). Methane emissions in East Asia for 2000–2011 estimated using an atmospheric Bayesian inversion. J. Geophys. Res. Atmos..

[13] US Energy Information Administration. China - international energy data and analysis. *eia Beta* https://www.eia.gov/beta/international/analysis.cfm?iso=CHN. Last access: 19 Mar 2018 (2015).

[14] International Energy Agency. Coal mine methane in China: a budding asset with the potential to bloom. IEA information paper. *IEA* https://www.iea.org/publications/freepublications/publication/china_cmm_report.pdf. Last access: 19 Mar 2018 (2009).

[15] US Environmental Protection Agency, Coalbed Methane Outreach Program. Energy markets in China and the outlook for CMM project development in Anhui, Chongqing, Henan, Inner Mongolia, and Guizhou provinces. *U.S. EPA* https://www.epa.gov/sites/production/files/2016-03/documents/2014_coalchinaenergymarket_fullreport.pdf. Last access: 19 Mar 2018 (2015).

[16] US Environmental Protection Agency, Coalbed Methane Outreach Program. Legal and regulatory status of CMM ownership in key countries: considerations for decision makers. *U.S. EPA* https://www.epa.gov/cmop/legal-and-regulatory-status-cmm-ownership-key-countries. Last access: 19 Mar 2018 (2014).

[17] Cashdollar KL, Zlochower IA, Green GM, Thomas RA, Hertzberg M (2000). Flammability of methane, propane, and hydrogen gases. J. Loss Prev. Process Ind..

[18] Huang, S. Current situations of CBM/CMM recovery and utilization & methane emission reduction in China. In *Global Methane Initiative Expo*. Vancouver, Canada., 12–15 (2013).

[19] Kuze A (2016). Update on GOSAT TANSO-FTS performance, operations, and data products after more than 6 years in space. Atmos. Meas. Tech..

[20] Dlugokencky, E. Global greenhouse gas reference network: trends in atmospheric methane. *NOAA Global Monitoring Division *www.esrl.noaa.gov/gmd/ccgg/trends_ch4/. Last access: 19 Mar 2018 (2017).

[21] Nisbet EG, Dlugokencky EJ, Bousquet P (2014). Methane on the rise—again. Science.

[22] Patra PK (2016). Regional methane emission estimation based on observed atmospheric concentrations (2002–2012). J. Meteorol. Soc. Jpn..

[23] Parker RJ (2018). Evaluating year-to-year anomalies in tropical wetland methane emissions using satellite CH_4_ observations. Remote Sens. Environ..

[24] Crevoisier C (2013). The 2007–2011 evolution of tropical methane in the mid-troposphere as seen from space by MetOp-A/IASI. Atmos. Chem. Phys..

[25] Bruhwiler LM (2017). U.S. CH_4_ emissions from oil and gas production: have recent large increases been detected?. J. Geophys. Res. Atmos..

[26] Kirschke S (2013). Three decades of global methane sources and sinks. Nat. Geosci..

[27] Saunois M (2016). The global methane budget 2000–2012. Earth Syst. Sci. Data.

[28] Ganesan AL (2017). Atmospheric observations show accurate reporting and little growth in India’s methane emissions. Nat. Commun..

[29] US Department of Agriculture Foreign Agricultural Service. Rice: world markets and trade, (Washington, DC). *USDA* http://usda.mannlib.cornell.edu/usda/fas/grain-market//2010s/2017/grain-market-10-12-2017.pdf. Last access: 19 Mar 2018 (2017).

[30] US Department of Agriculture Foreign Agricultural Service. Livestock and poultry: World markets and trade, (Washington, DC). *USDA* https://apps.fas.usda.gov/psdonline/circulars/livestock_poultry.pdf Last access: 19 Mar 2018 (2017).

[31] Inouye, A. China: *Dairy and products semi-annual - 2017*. Global Agricultural Information Network report CH17031. (US Department of Agriculture, Foreign Agricultural Service, 2017).

[32] US Environmental Protection Agency, Coalbed Methane Outreach Program. Coal mine methane country profiles. *Global Methane Initiative* http://www.globalmethane.org/tools-resources/coal_overview.aspx. Last access: 19 Mar 2018 (2015).

[33] Guan D, Liu Z, Geng Y, Lindner S, Hubacek K (2012). The gigatonne gap in China’s carbon dioxide inventories. Nat. Clim. Change.

[34] Korsbakken JI, Peters GP, Andrew RM (2016). Uncertainties around reductions in China’s coal use and CO_2_ emissions. Nat. Clim. Change.

[35] National Development and Reform Commission of the People’s Republic of China. Second National Communication on Climate Change of The People’s Republic of China. *UNFCCC* https://unfccc.int/resource/docs/natc/chnnc2e.pdf. Last access: 20 July 2018 (2012).

[36] Butz A, Hasekamp OP, Frankenberg C, Vidot J, Aben I (2010). CH_4_ retrievals from space-based solar backscatter measurements: Performance evaluation against simulated aerosol and cirrus loaded scenes. J. Geophys. Res. Atmos..

[37] Schepers D (2012). Methane retrievals from greenhouse gases observing satellite (GOSAT) shortwave infrared measurements: Performance comparison of proxy and physics retrieval algorithms. J. Geophys. Res. Atmos..

[38] Hasekamp, O. et al. Algorithm Theoretical Basis Document for the RemoTeC XCH_4_ Proxy Product v2.3.8. ESA Climate Change Initiative (CCI). *ESA* http://www.esa-ghg-cci.org/?q=webfm_send/367. Last access: 9 Jul 2018 (2016).

[39] Wunch D (2011). The total carbon column observing network. Philos. Trans. R. Soc. Lond. A Math. Phys. Eng. Sci..

[40] Monteil G (2013). Comparison of CH_4_ inversions based on 15 months of GOSAT and SCIAMACHY observations. J. Geophys. Res. Atmos..

[41] Fraser A (2013). Estimating regional methane surface fluxes: the relative importance of surface and GOSAT mole fraction measurements. Atmos. Chem. Phys..

[42] Turner AJ (2015). Estimating global and North American methane emissions with high spatial resolution using GOSAT satellite data. Atmos. Chem. Phys..

[43] Pickett-Heaps CA (2011). Magnitude and seasonality of wetland methane emissions from the Hudson Bay Lowlands (Canada). Atmos. Chem. Phys..

[44] European Commission Joint Research Centre. Global emissions EDGAR v4.2. *European Commission* http://edgar.jrc.ec.europa.eu/overview.php?v=42. Last access: 19 Mar 2018 (2017).

[45] Giglio L, Randerson JT, van der Werf GR (2013). Analysis of daily, monthly, and annual burned area using the fourth-generation global fire emissions database (GFED4). J. Geophys. Res. Biogeosci..

[46] van der Werf GR (2017). Global fire emissions estimates during 1997–2016. Earth Syst. Sci. Data.

[47] Baker, D. F. et al. TransCom 3 inversion intercomparison: Impact of transport model errors on the interannual variability of regional CO_2_ fluxes, 1988–2003. *Global. Biogeochem. Cycles* **20** GB1002, (2006).

[48] Wecht KJ, Jacob DJ, Frankenberg C, Jiang Z, Blake DR (2014). Mapping of North American methane emissions with high spatial resolution by inversion of SCIAMACHY satellite data. J. Geophys. Res. Atmos..

[49] Turner, A. J., Frankenberg, C., Wennberg, P. O. & Jacob, D. J. Ambiguity in the causes for decadal trends in atmospheric methane and hydroxyl. *Proc. Natl Acad. Sci. USA* **114**, 5367–5372 (2017). 10.1073/pnas.1616020114 PMC5448216 28416668

[50] Zhang B, Chen G, Li J, Tao L (2014). Methane emissions of energy activities in China 1980–2007. Renew. Sustain. Energy Rev..

[51] Schwietzke S, Griffin WM, Matthews HS, Bruhwiler LMP (2014). Global bottom-up fossil fuel fugitive methane and ethane emissions inventory for atmospheric modeling. ACS Sustain. Chem. Eng..

[52] Wecht, K. J., Jacob, D. J., Frankenberg, C., Jiang, Z. & Blake, D. R. Mapping of North American methane emissions with high spatial resolution by inversion of SCIAMACHY satellite data. *J. Geophys. Res. Atmos.* **119**, 7741–7756 (2014).

[53] United National Framework Convention on Climate Change. Greenhouse gas inventory data—detailed data by party. *UNFCCC *http://di.unfccc.int/detailed_data_by_party. Last access: 20 July 2018 (2018).

[54] Zhang, B., Chen, G., Li, J. & Tao, L. Methane emissions of energy activities in China 1980–2007. *Renew. Sustain. Energy Rev.* **29**, 11–21 (2014).

[55] Schwietzke, S., Griffin, W. M., Matthews, H. S. & Bruhwiler, L. M. P. Global bottom-up fossil fuel fugitive methane and ethane emissions inventory for atmospheric modeling. *ACS Sustain. Chem. Eng.* **2**, 1992–2001 (2014).

