# Supplementary material for: China’s coal mine methane regulations have not curbed growing emissions
Source: Nat Commun. 2019 Jan 29;10:303. doi: 10.1038/s41467-018-07891-7 (PMC6351523; doi:10.1038/s41467-018-07891-7)
Supplement: Supplementary file 1 — Supplementary Information [file 41467_2018_7891_MOESM1_ESM.pdf]

Supplemental information to "China's coal mine methane regulations have not curbed growing emissions"

Scot M. Miller[1,2], Anna M. Michalak[1], Robert G. Detmers[3], Otto P. Hasekamp[3], Lori M.P. Bruhwiler[4] & Stefan Schwietzke[4,5,6]

1. Department of Global Ecology, Carnegie Institution for Science, 260 Panama St., Stanford, CA 94305 USA.
2. Now at the Department of Environmental Health and Engineering, Johns Hopkins University, 3400 N. Charles Street, Baltimore, MD 21218 USA.
3. SRON, Netherlands Institute for Space Research, 3584 CA Utrecht, Netherlands.
4. Global Monitoring Division, National Oceanic and Atmospheric Administration, 325 Broadway R/GMD 1, Boulder, CO 80305 USA.
5. Cooperative Institute for Research in Environmental Sciences, University of Colorado Boulder, 216 UCB, Boulder, CO 80309 USA.
6. Now at Environmental Defense Fund, 2060 Broadway, Boulder, CO 80302 USA.

Supplementary Figures

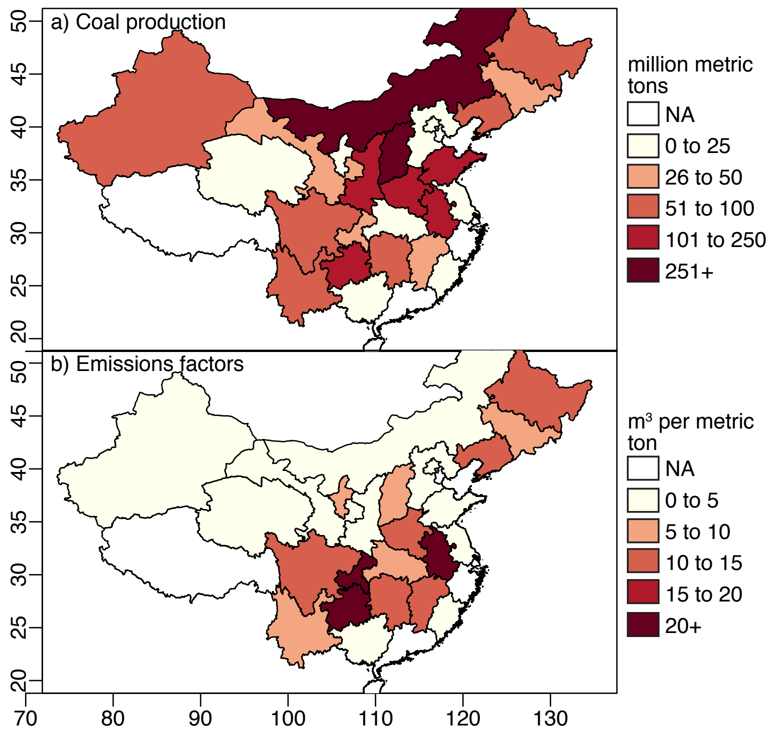

Supplementary Figure 1: Coal production and emissions by province in China. Estimates of coal production (a) and coal emissions factors (b) for 2010 from Zhu et al. (56). The estimated CH_4 emissions in Supplementary Figure 3b are high in regions with large coal production and high emissions factors.

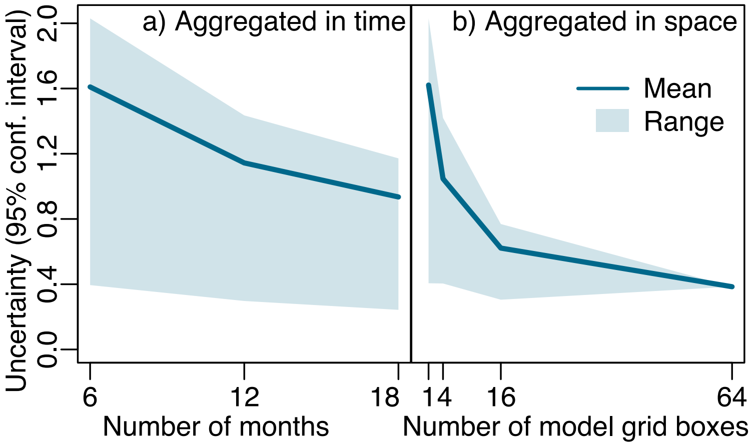

Supplementary Figure 2: Emissions uncertainties at aggregated regions and times. Uncertainties in the estimated emissions at increasingly aggregated time scales (a) and at increasingly aggregated spatial scales (b). The estimated emissions are highly uncertain for individual model grid boxes and for 6-month time periods, but those uncertainties decrease for larger regions and longer time periods. Note that the y-axis is in units of emissions scaling factor, the quantity estimated by the inverse model (e.g., $\boldsymbol{\lambda}$). Furthermore, the uncertainties are 95% confidence intervals, and the 95% confidence interval (i.e., 1.95σ) on the prior scaling factors is 1.95 for a single model grid box in Asia.

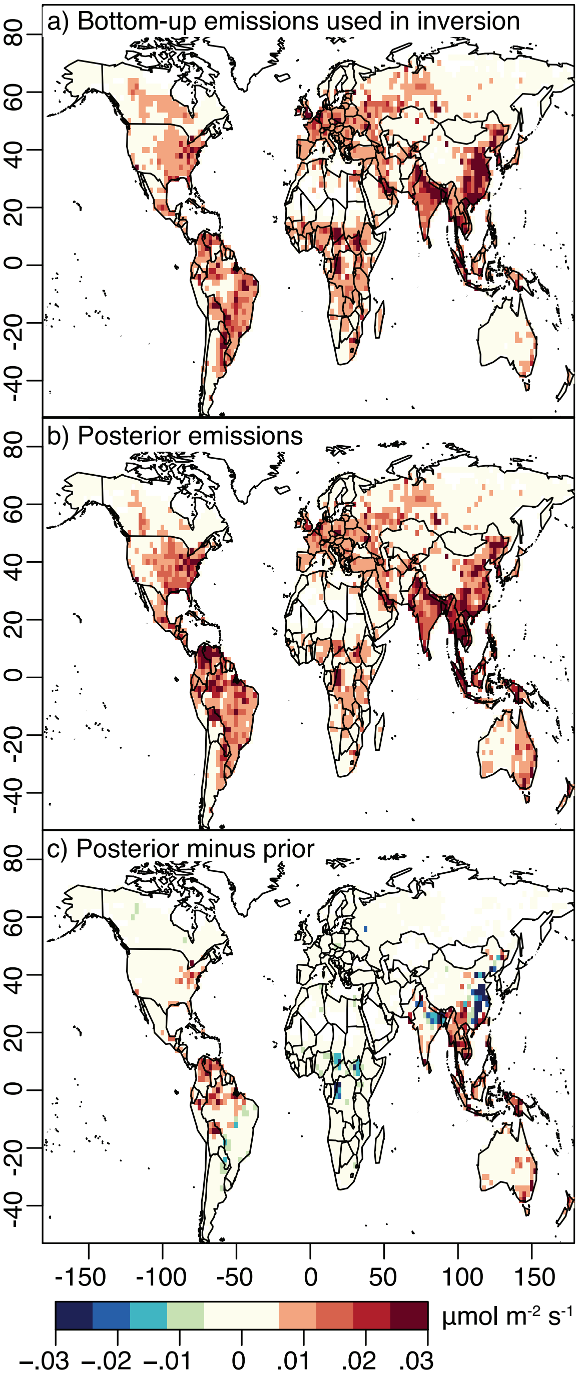

Supplementary Figure 3: Prior and posterior CH_4 emissions estimates. The emissions inventories used in the inverse model (a), the posterior emissions estimate (b), and the differences between these two estimates (c). The global maps shown are the averages of all time periods (2010–2015). We estimate lower emissions in China and regions of Africa and higher emissions in tropical Asia and South America. This result is broadly consistent with several existing inverse modeling studies (e.g., 57; 58; 59).

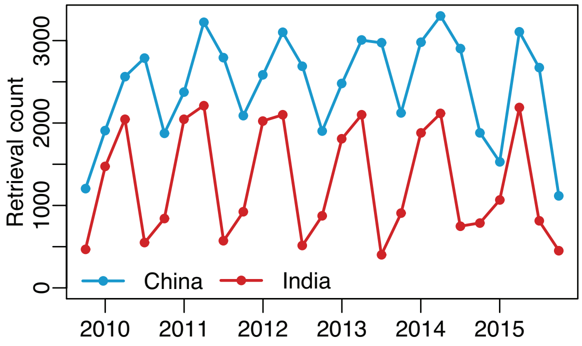

Supplementary Figure 4: Number of GOSAT observations per season. The number of nadir GOSAT retrievals without a negative quality control flag across India and China for each season between 2009 and 2015. The number of observations varies by season but does not show a year-to-year trend.

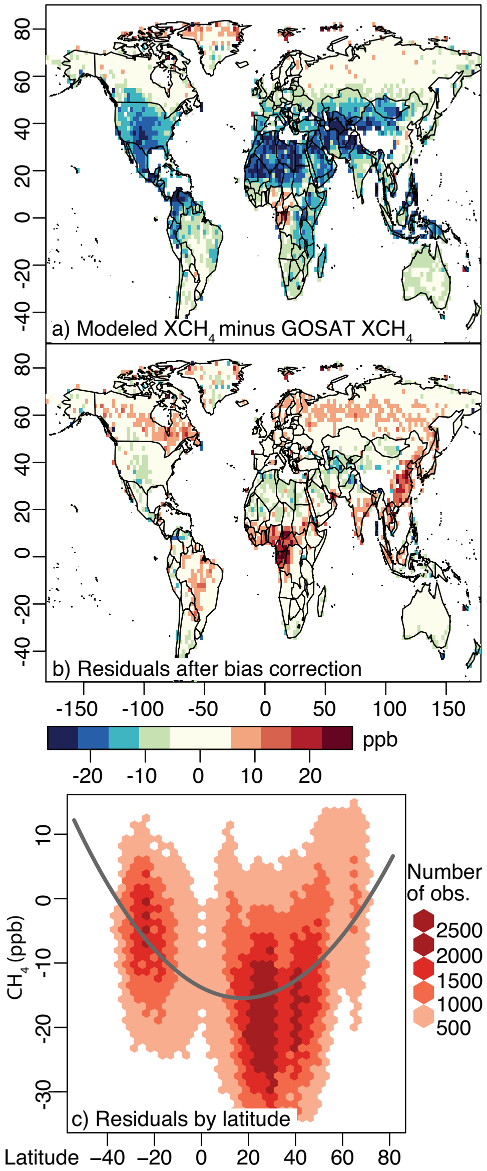

Supplementary Figure 5: Global model–data bias correction. Differences between GOSAT observations and modeled XCH_4 before a latitudinal bias correction (a), the model-data differences following this correction (b), and the bias plotted as a function of latitude (c). Darker colors in panel (c) indicate a greater number of observations, and the grey line is a fitted quadratic regression. The bias is much smaller after the correction (b) than before the correction (a).

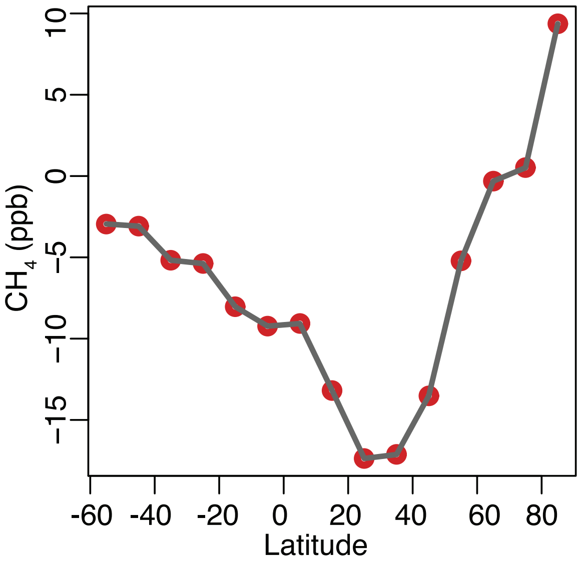

Supplementary Figure 6: The alternate method to correct the latitudinal bias. The two methods, in combination, provide a measure of uncertainty in the bias correction. In this alternate method, we average the model–data difference into 10° latitude bins and interpolate between those points, as shown in this figure. The emissions estimated in this study are similar irrespective of the correction method employed (Supplementary Note 2).

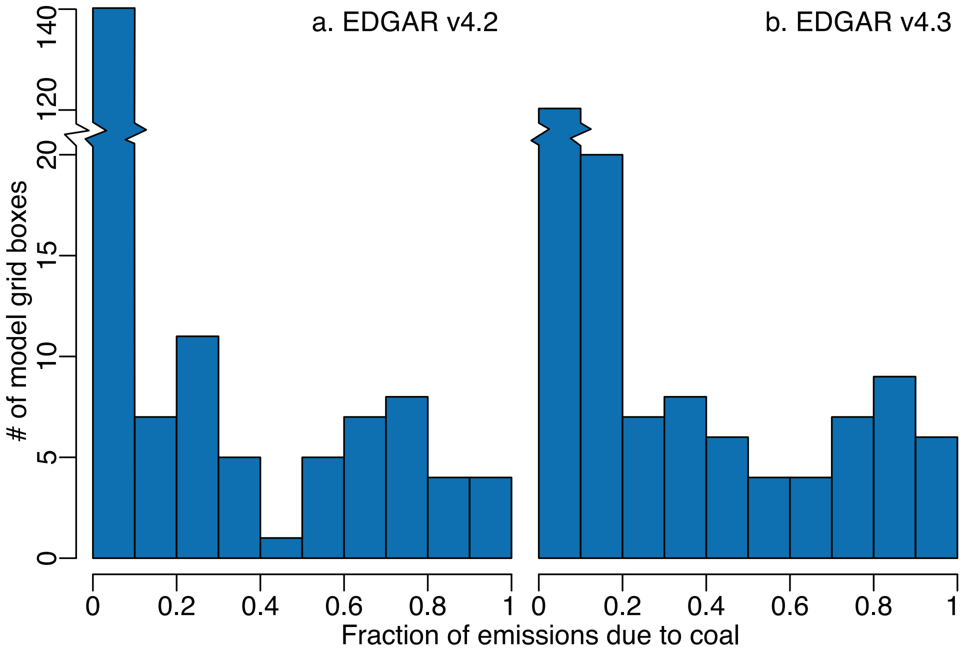

Supplementary Figure 7: CH_4 emissions from coal in EDGAR v4.2 and v4.3. Coal as a fraction of total emissions in two different versions of the EDGAR emissions inventory. We compare the year 2008, the year used in the prior emissions estimate. Each bar indicates the number of model grid boxes in China with a certain fraction of emissions due to coal. The older inventory version v4.2 contains more grid boxes with a small fraction of emissions due to coal. It also contains fewer grid boxes in which a high fraction (>80%) of emissions are due to coal. Note that the gridded version of v4.3 includes all emissions related to fossil fuel extraction within a single category (e.g., oil, gas, and coal). EDGAR v4.2, by contrast, has a specific emissions category for coal mining emissions.

Supplementary Notes

Supplementary Note 1

This note describes the RemoTeC proxy retrieval. The analysis presented in this paper utilizes the RemoTeC proxy retrieval (e.g., 60). The proxy method incorporates modeled and observed CO_2. Differences between the CO_2 observations and model are used to correct GOSAT total column methane (XCH_4) for light path modifications (e.g., aerosols, clouds) (e.g., 61):

$$XCH_4 = \frac{XCO_2}{X^*CO_2} X^*CH_4 \tag{S1}$$

where X^*CO_2 and X^*CH_4 are the retrieved CO_2 and CH_4 mole fractions from GOSAT, XCO_2 is the modeled CO_2 estimate, and XCH_4 is the final estimate. The RemoTeC retrieval uses atmospheric mixing ratios from CarbonTracker CO_2 as the model estimate (62). Any differences between the model and retrieval are assumed to be due to light path modifications, so this correction is dependent upon the accuracy of the CarbonTracker product. Also note that both gases are retrieved under the assumption of a non-scattering atmosphere. Furthermore, the proxy method can retrieve more data points relative to a full physics method because the former approach can tolerate greater cloud and aerosol contamination than the latter (e.g., 63). One study lists precision and accuracy statistics for the proxy method, and they are similar to the full physics method (60). That study compares the GOSAT proxy retrievals against TCCON (Total Carbon Column Observing Network). The retrieval bias ranges from -0.312% to 0.421% (~ -6 to 8 ppb), and single-sounding precision is $\sim$17 ppb (standard deviation) (60).

The number of nadir retrievals does not show a trend over the study period (Supplementary Figure 4). Any multi-year trends in the availability of data could result in spurious trends in the estimated emissions. The availability of observations does, however, vary by season. The number of retrievals over India is highest in winter and spring and is lowest in summer during the monsoon. By contrast, the number of retrievals over China is highest in spring and lowest in fall.

Note that we do not use GOSAT observations over Antarctica and over high altitude regions (surface elevation >3000m). The signal-to-noise ratio over Antarctica is small, and any errors or biases in these observations could substantially bias our inferences for the southern hemisphere. Observations over high altitude, mountainous regions are also challenging to model.

Supplementary Note 2

This note describes the model–data bias correction. The GOSAT observations show a latitude-dependent difference when compared to modeled XCH_4 (using existing, bottom-up emissions estimates). Supplementary Figures 5a and 5c illustrate this bias; it has the same shape as the model–data bias described in two previous studies (64; 57). The observations are an overestimate relative to the model in the northern hemisphere tropics and mid-latitudes (centered around 20° N). By contrast, the observations are an underestimate relative to the model at high latitudes.

The model–data bias could be caused by a number of factors, but circumstantial evidence points to a model–data differences in the stratosphere. Turner et al. (57) examine and diagnose this difference in detail relative to existing in situ and TCCON observations. Global GEOS-Chem simulations show a minimal overall bias ($\sim$4ppb) and no latitudinal bias when compared to HIPPO (HIAPER Pole-to-Pole Observations) observations. The authors further compare global GEOS-Chem outputs against TCCON and report an R^2 of 0.82 (prior model) and 0.83 (posterior model), and mean biases of 6.4ppb and 8.1ppb, respectively. These biases are generally smaller than the latitudinal differences between GOSAT and GEOS-Chem (e.g., Supplementary Figure 5).

The Turner et al. (57) analysis, and the fact that the GEOS-Chem–GOSAT bias is largest in very remote regions like the South Pole, indicates that this bias is unlikely to be due to the emissions inventories. Rather, Turner et al. (57) speculate that this bias is either due to biases in GEOS-Chem in the stratosphere or due to a latitude-dependent bias in GOSAT. Fraser et al. (64) further speculate that the model–GOSAT differences could be due to cirrus clouds, sensitivity of the satellite to zenith angle, and/or errors in modeled CO_2 used in the proxy retrieval.

We correct the GOSAT observations before running the inverse model to account for this bias. This correction should not change regional patterns in emissions (e.g., patterns across India or across China); the correction used here adjusts large-scale, latitudinal gradients, not regional-scale variability in the GOSAT observations. Specifically, we fit a quadratic regression to the model–data residuals as a function of latitude (Supplementary Figure 5c). We then add these fitted values to the GOSAT observations. This approach is the same as that employed by an earlier study (57). Supplementary Figure 5b displays the model–data residuals after applying the correction. The resulting residuals exceed 20 ppb in some regions but do not show a consistent global bias.

We also explore a second, alternate method to correct the latitude-bias; this second method serves as a check on the robustness or uncertainty in the impact of the first approach described above. In this alternate correction, we average the bias in latitude bands that are 10° in width. We then interpolate between those bands using linear interpolation, shown in Supplementary Figure 6. These interpolated values are used as the bias correction. We re-run the inverse model using observations that have been corrected with this alternate method. The annual CH_4 budget for India varies by up to ±0.1 Tg CH_4 depending upon the bias correction used, and the budget for China varies by up to ±0.6 Tg CH_4, small numbers relative to the total budgets. Thus, the choice of bias correction does not have a noticeable effect on the estimated trend (less than 0.1 Tg CH_4 yr^{-1} for either India or China).

Supplementary Note 3

This note provides additional detail on the GEOS-Chem model. We employ version v11-01 of the GEOS-Chem model in this study. This version of GEOS-Chem uses a new emissions module, the Harvard-NASA Emissions Component or HEMCO, and this module makes it relatively straightforward to modify the emissions input into GEOS-Chem (65). We run these GEOS-Chem simulations in a tagged tracer format for the inverse model, discussed in detail in the next section.

All of the GEOS-Chem simulations here use GEOS meteorology. Furthermore, we use GEOS meteorology to drive the wetland emissions model (e.g., soil temperature and soil moisture). This setup mirrors that of previous GEOS-Chem studies of CH_4 (e.g., 57; 66; 67; 68). GEOS-Chem uses two different versions of GEOS meteorology. GEOS version 5 simulations are available through May 31, 2013. By contrast, GEOS version 5 forward processing (GEOS-5 FP) simulations are available beginning in April 2012. These fields are provided by the Global Modeling and Assimilation Office (GMAO) at NASA Goddard Space Flight Center. In this study, we use GEOS-5 meteorology for model runs from 2009 to 2012 and GEOS-FP meteorology for model runs from 2013-2015.

We explore uncertainties in the meteorology by comparing the estimated emissions trend for China during 2010-2012 against the trend during 2013-2015. We use GEOS-5 meteorology during the former time period and GEOS-FP during the latter. This comparison therefore indicates the sensitivity of the trend to the meteorology product (GEOS-5 versus GEOS-FP). The estimated trend is consistent between the two time periods that use different GEOS meteorology products; we estimate an emissions trend of 1.4 ± 3.58 Tg CH_4 yr^{-1} for China during 2010-2012 and a trend of 1.5 ± 0.34 Tg CH_4 yr^{-1} for 2013-2015. Note that the overall 6-year

trend (1.1 ± 0.4) is slightly less than the trend during either three year window – due to a slight drop in estimated emissions in 2013 at the beginning of the GEOS-FP model runs. In contrast to China, the estimated 6-year trend in emissions from India is dominated by an anomalously high emissions estimate during 2014. The estimated trend is therefore highly uncertain, and it is difficult to compare or contrast the trend from 2010-2012 against that estimated for 2013-2015.

Supplementary Note 4

This note provides an overview of the inverse modeling setup. This section outlines the specific inverse modeling setup employed in this study. The analysis in this study depends upon atmospheric transport model runs from the GEOS-Chem model, and we use a tagged tracer setup for these atmospheric model runs. In other words, we run the transport model once for each model grid box within Asia and each 6-month time period. Outside of Asia, we run the transport model once for each TransCom region (The Atmospheric Tracer Transport Model Intercomparison Project) (e.g., 69) and each 6-month time period. This setup makes it straightforward to compute a posterior covariance matrix, which forms the basis of the uncertainty bounds on the final posterior emissions estimate.

It is important to note that we use anthropogenic emissions from EDGAR (version 4.2) for the year 2008 as part of the prior emissions model. As a result of this setup, the anthropogenic emissions inventory used in the inversion is time invariant. This setup ensures that any trend in the posterior emissions reflects a trend in the observations and is not due to a trend in the EDGAR emissions inventory. By contrast, the wetland and biomass burning emission inventories do vary with time – to account for irregular, year-to-year variations in wildfires and soil freeze/thaw. These sources, however, do not show a notable trend across China and India for the study period.

These emissions and transport model runs become inputs into the inverse model. We estimate the emissions in the inverse model by minimizing the following cost function:

$$\begin{aligned} L \; = \; & \left(\boldsymbol{z} - \sum_{i}^{p} \boldsymbol{\lambda}_i h(\boldsymbol{s})_i \right)^T \mathbf{R}^{-1} \left(\boldsymbol{z} - \sum_{i}^{p} \boldsymbol{\lambda}_i h(\boldsymbol{s})_i \right) \\ & + (\boldsymbol{\lambda} - \boldsymbol{\mu})^T \mathbf{Q}^{-1} (\boldsymbol{\lambda} - \boldsymbol{\mu}) \end{aligned} \quad \text{(S2)}$$

where $\boldsymbol{z}$ represents the observations (dimensions $n \times 1$). Note that we do not average the GOSAT observations before using them in the inverse model. In addition, the covariance matrices, $\mathbf{R}$ ($n \times n$) and $\mathbf{Q}$ ($p \times p$), describe uncertainties in the observations/model and in the prior scaling factors, respectively. The variable $h(\boldsymbol{s})_i$ ($n \times 1$) represents the modeled XCH_4 from emissions in region and time period i using bottom-up emissions estimates; $\boldsymbol{s}$ represents the bottom-up emissions and $h()$ the atmospheric transport model, in this case the GEOS-Chem model.

We estimate a set of scaling factors in the inverse model that scale the bottom-up emissions; the vector $\boldsymbol{\lambda}$ represents these estimated scaling factors ($p \times 1$). Specifically, $\boldsymbol{\lambda}_i$ is the scaling factor for an individual model grid box or TransCom region for a 6-month time period. The inverse model implemented here is Bayesian and includes a prior estimate of the scaling factors, denoted $\boldsymbol{\mu}$ ($p \times 1$). In the setup here, the prior estimate of the scaling factors is constant for each 6-month time period of the inverse model, unlike the posterior scaling factors ($\boldsymbol{\lambda}$) which vary from one model grid box to another. Furthermore, this prior value is an unknown quantity in the inverse model implemented here. This setup is different from many Bayesian inverse models that fix the prior scaling factors at one, and this setup ensures that the prior estimate is unbiased relative to the posterior estimate, a statistical assumption of the inverse model. For example, the emissions inventories in the inverse model likely have an overall bias across China (e.g., 57; 58). We solve for $\boldsymbol{\mu}$ as part of the inverse model, thus guaranteeing that the

prior model is unbiased relative to the GOSAT observations. Hence, we minimize Eq. S2 with respect to both $\boldsymbol{\mu}$ and $\boldsymbol{\lambda}$.

In the setup here, we estimate both the emissions and the covariance matrix parameters by minimizing Eq. S2. We minimize this cost function (Eq. S2) using an expectation maximization algorithm (e.g., 70; 71). We first make an initial guess for the covariance matrix parameters and then estimate the emissions by finding the minimum of Eq. S2 with respect to $\boldsymbol{\lambda}$ and $\boldsymbol{\mu}$. We then estimate the covariance matrix parameters, using the previously-estimated emissions and the associated posterior covariance matrix (discussed below) (e.g., 70). We alternate between estimating covariance matrix parameters and the emissions until all of the estimated values converge on stable estimates. This process usually requires approximately three or four iterations.

In this particular setup, we further require that the estimated scaling factors ($\boldsymbol{\lambda}$) be non-negative. We therefore subsequently enforce non-negativity using Lagrange multipliers (72).

As a final step, we estimate uncertainties in the estimated scaling factors ($\boldsymbol{\lambda}$) and hence uncertainties in the estimated emissions. To accomplish this task, we estimate a posterior covariance matrix for $\boldsymbol{\lambda}$ (denoted $\mathbf{V}_{\boldsymbol{\lambda}}$, dimensions $p \times p$). We calculate this matrix by computing the inverse of the Hessian of Eq. S2. In this case, the Hessian is the second derivative of Eq. S2 with respect to $\boldsymbol{\lambda}$ and $\boldsymbol{\mu}$.

Note that we estimate the emissions using an 18-month moving window, and we re-run the inverse model (including covariance estimation) for each time window. The scaling factors ($\boldsymbol{\lambda}$) estimated by the inversion vary every 6 months, so the 18-month window encapsulates three sets of scaling factors. We discard the first 6-month period as a spin-up period, save the output for the middle 6-month period, and discard the last 6-month period. We then shift the moving window forward by 6-months and run the inverse model again, using the procedure described above.

Supplementary Note 5

This note describes the covariance matrices used in the inverse model. The inverse model requires two covariances matrices, one that describes errors in the atmospheric model and observations ($\mathbf{R}$) and one that describes errors in the prior scaling factors ($\mathbf{Q}$). The diagonal elements of these matrices describe error variances. By contrast, the off-diagonal elements describe spatial and temporal covariances among the errors, and we include off-diagonal elements in both $\mathbf{R}$ and $\mathbf{Q}$ for the setup here. We model the off-diagonal elements of both covariance matrices using a spherical covariance model with a nugget (e.g., 73). This model, as implemented here, contains four parameters. The nugget variance of the model represents white noise errors – errors that are not correlated in space and/or time. The model also contains a sill variance, and this parameter represents errors that are correlated in space and/or time. Lastly, this model contains a decorrelation length and a decorrelation time. These parameters indicate the spatial and temporal scales over which the errors are correlated. Unlike other covariance models, a spherical model decays to zero beyond the decorrelation length and decorrelation time. This feature makes it possible to formulate the covariance matrices as sparse matrices, saving on both memory and computing time.

We structure the $\mathbf{R}$ matrix as a block diagonal matrix. $\mathbf{R}$ has a large number of rows and columns (an average of ~270,000 rows/columns, depending upon the time period), and it is not computationally feasible to invert a very large, non-sparse $\mathbf{R}$ matrix. To make these calculations computationally tractable, we formulate $\mathbf{R}$ as a block diagonal matrix where each block is a different continent, and each block has an average of ~45,000 observations. As a result of this setup, we assume that the model and observational errors are spatially and temporally correlated within each individual continent but not among different continents.

We estimate several elements of $\mathbf{R}$ using Eq. S2 – both the nugget variance and the sill variance. We estimate a constant global value for each variance term, but we allow this variance term to vary among different time periods of the inverse model. We estimate average values of $(8.2 \text{ ppb})^2$ and $(17.5 \text{ pbb})^2$, respectively (averaged across all time periods of the inverse model).

Note that some of the covariance matrix parameters are difficult to estimate using Eq. S2; the value of this cost function is sensitive to the values of certain covariance matrix parameters but is relatively insensitive to the value of other parameters. As a result, we use expert judgement to estimate the decorrelation length and decorrelation time in $\mathbf{R}$. We estimate 2000 km for the decorrelation length in $\mathbf{R}$ (i.e., the distance at which correlations are assumed to reach zero) and 30 days for the decorrelation time.

The covariance matrix $\mathbf{Q}$ also contains both diagonal and off-diagonal elements. Equation S2 would not converge on values for $\mathbf{Q}$, so we estimate these values using expert judgement. We estimate values of $(0.75)^2$ for the sill variance in $\mathbf{Q}$ in TransCom regions and $(1)^2$ for the sill variance in $\mathbf{Q}$ for the model grid boxes in Asia (in units of (scaling factor)^2). Note that we set a lower uncertainty for the TransCom regions because these regions are larger in area than the individual model grid boxes in Asia, and variances typically decrease when averaged over larger and larger areas. We include off-diagonal elements in $\mathbf{Q}$ that account for spatial covariances among the estimated scaling factors. We estimate a decorrelation length in $\mathbf{Q}$ of 500km. This length is similar to or slightly smaller than the size of coal mining or agricultural provinces in China. We further assume that uncertainties in the prior scaling factors ($\boldsymbol{\mu}$) are uncorrelated from one 6-month time period to the next, and we do not include a nugget variance in $\mathbf{Q}$.

Supplementary Note 6

This note provides additional detail on patterns in the estimated emissions. The posterior emissions have a different magnitude and different patterns relative to existing bottom-up estimates. Supplementary Figure 3a displays the bottom-up emissions estimates used as inputs in the inverse model, averaged over all years of this study. Supplementary Figures 3b-c, by contrast, show the posterior emissions estimate and the differences from the bottom-up estimates (Note that Supplementary Figure 3b is identical to Figure 3 in the main article.). The inverse model decreases emissions across northern India and much of China relative to the EDGAR v4.2 inventory, the anthropogenic inventory used in the inverse model. This result is consistent with several existing inverse modeling estimates (e.g., 57; 58; 59). Note that the EDGAR v4.3 inventory (discussed in the main manuscript) allocates smaller emissions across much of this region relative to v4.2 (74). The emissions estimated in this study are also higher across tropical South America and tropical Asia relative to the bottom-up emissions estimates, a result that is consistent with previous GOSAT-based inverse models (e.g., 57).

The estimated CH_4 emissions for China are highest in regions with large coal production and regions with high coal CH_4 content (e.g., high reported emissions factors). Supplementary Figure 1a displays coal production in China by province and Supplementary Figure 1b shows estimated coal mining emissions factors, as reported by Zhu et al. (56). Provinces like Shanxi, Guizhou, and Anhui have high coal production and high estimated emissions factors. These regions are also associated with large CH_4 emissions in the inverse modeling estimate (Supplementary Figure 3b).

Supplementary Note 7

This note give additional context on the uncertainties in the estimated emissions. The uncertainties in any single model grid box for any single time period is high (Supplementary Figure 2); the inverse model does not provide a robust constraint on emissions from individual grid boxes, and differences in the emissions from one grid box to another are usually within the

range of uncertainties. By contrast, we have much greater confidence in annual or multi-year emissions totals for entire countries. The GOSAT observations provide a stronger constraint on emissions at these aggregate space-time scales.

Supplementary Note 8

This note places the results of this study in the context of global observations of methane isotopes. Existing studies that interpret CH_4 isotopes ($\delta^{13}C$) provide a few possible explanations for global trends in CH_4 emissions. Several studies argue that total global fossil fuel CH_4 emissions may have remained relatively flat in the last decade and have not not been driving the global CH_4 increase since 2007 (75; 76). Another study, by contrast, argues that biomass burning CH_4 emissions have decreased while fossil fuel emissions have increased (77). The trends estimated in this study are not inconsistent with either explanation. For example, Schwietzke et al. (78) argue that decreasing natural gas CH_4 emissions at global scale have been compensated by increasing coal emissions to produce a flat global trend in total fossil fuel CH_4 emissions (Figure S10 in Schwietzke et al. (78)), and these trends are qualitatively valid even under decreasing biomass burning CH_4 emissions. Natural gas operations, they argue, have become more efficient over time, and leak rates of decreased from a global average of 8% to 2% over the past 30 years. By contrast, it is more likely that emissions factors from coal operations have remained unchanged during the same time period, and total coal CH_4 emissions have increased as total natural gas CH_4 emissions have declined. Furthermore, the increase in coal CH_4 emissions from China for 2010-2012 estimated in this study is less than the global coal emissions increase estimated in Schwietzke et al. (78) for the same time period. Hence, the emissions estimated here for China are not inconsistent with trends in atmospheric $\delta^{13}C$ observations. Furthermore, another study (79) points out that the isotopic signature of coal CH_4 emissions varies widely depending upon the coal type; more isotopic observations of coal CH_4 from diverse regions of the globe would help better constrain the contribution of these emissions to global trends in $\delta^{13}C$.

Supplementary Note 9

This note describes the effects of possible changes in the hydroxyl radical (OH). We find that potential changes on global OH are unlikely to have an effect on the overall results of this study. A handful of recent studies raise the possibility that OH may have changed in recent years (80; 81) while other studies argue that there is no evidence for recent changes in OH (82; 83). Possible changes in OH would yield a trend in CH_4 observations that is much smaller than the observed trend across eastern China. As a result, the majority of the XCH_4 trend across China is likely due to emissions, even if OH were changing. Specifically, Figure 1 indicates a trend in XCH_4 of 0.2 ppb per year in eastern and central China relative to sparsely populated upwind regions of western China and central Asia. One study predicts that the lifetime of CH_4 has been reduced by 4 months since the pre-industrial era (84). Air masses likely take less than 10 days to advect from central Asia to eastern China, and this change in the CH_4 lifetime would result in a net change in CH_4 of 0.09 ppb over 10 days for the present time relative to pre-industrial OH levels. This net change of 0.09ppb would be far smaller than the 0.2ppb annual trend across eastern China.

Supplementary Note 10

This note provides additional detail on the source attribution of estimated CH_4 emissions. We use several different pieces of information to attribute emissions to different source sectors, and one key piece of information is the fraction of emissions due to coal within each grid box of the

EDGAR inventory. We use this information to attribute the emissions estimated in this study to either coal mining or other source sectors. Any uncertainty in this fraction could propagate into uncertainty in the source attribution presented in this study. In this section of the SI, we evaluate the uncertainty in this fraction and discuss what effect this uncertainty may have on the source attribution presented in this paper.

One way to evaluate this uncertainty is to examine how this fraction (i.e., the fraction of emissions attributed to coal) differs among different versions of the EDGAR emissions inventory. Supplementary Figure 7 compares this fraction for EDGAR v4.2 versus v4.3. A large difference between these two versions would indicate high uncertainty in the source attribution and vice versa. Note the we use EDGAR v4.2 in the prior estimate of the emissions and use this version to attribute emissions to different source sectors.

We conclude that the source attribution to coal in this study may be conservative (i.e., too low); the attribution to coal is unlikely to be too high. Specifically, the fraction of emissions attributed to coal is similar between the two inventory versions with a few notable exceptions. Version 4.3 has fewer grid boxes with a low fraction of emissions due to coal (<10%) and has more boxes with a high fraction of emissions due to coal (>80%). As a result, the source attribution presented in this study assigns a smaller fraction of emissions to coal than if we had used EDGAR v4.3.

It is important to note that the gridded version of EDGAR v4.3, unlike v4.2, does not include separate categories for emissions from coal mining, oil drilling, and gas drilling; they are combined into a single emissions category (74). However, oil and gas are a small component of China's energy portfolio, and oil and gas CH_4 emissions in China are on the order of 10% of coal mining emissions according to EDGAR v4.2. Hence, the comparison between EDGAR v4.2 and v4.3 in Supplementary Figure 7 is not precise, though the different emissions categories are relatively similar.

Supplementary References

[56] Zhu, T., Bian, W., Zhang, S., Di, P. & Nie, B. An improved approach to estimate methane emissions from coal mining in China. *Environmental Science & Technology* **51**, 12072–12080 (2017). PMID: 28956434.

[57] Turner, A. J. *et al.* Estimating global and North American methane emissions with high spatial resolution using GOSAT satellite data. *Atmospheric Chemistry and Physics* **15**, 7049–7069 (2015).

[58] Bergamaschi, P. *et al.* Atmospheric CH_4 in the first decade of the 21st century: Inverse modeling analysis using SCIAMACHY satellite retrievals and NOAA surface measurements. *Journal of Geophysical Research: Atmospheres* **118**, 7350–7369 (2013).

[59] Bruhwiler, L. *et al.* Carbontracker-CH_4: an assimilation system for estimating emissions of atmospheric methane. *Atmospheric Chemistry and Physics* **14**, 8269–8293 (2014).

[60] Schepers, D. *et al.* Methane retrievals from Greenhouse Gases Observing Satellite (GOSAT) shortwave infrared measurements: Performance comparison of proxy and physics retrieval algorithms. *Journal of Geophysical Research: Atmospheres* **117** (2012). D10307.

[61] Frankenberg, C. *et al.* Satellite chartography of atmospheric methane from SCIAMACHY on board ENVISAT: Analysis of the years 2003 and 2004. *Journal of Geophysical Research: Atmospheres* **111** (2006). D07303.

[62] Peters, W. *et al.* An atmospheric perspective on North American carbon dioxide exchange: Carbontracker. *Proceedings of the National Academy of Sciences* **104**, 18925–18930 (2007).

[63] Monteil, G. *et al.* Comparison of CH_4 inversions based on 15 months of GOSAT and SCIAMACHY observations. *Journal of Geophysical Research: Atmospheres* **118**, 11,807–11,823 (2013).

[64] Fraser, A. *et al.* Estimating regional methane surface fluxes: the relative importance of surface and GOSAT mole fraction measurements. *Atmospheric Chemistry and Physics* **13**, 5697–5713 (2013).

[65] Keller, C. A. *et al.* HEMCO v1.0: a versatile, ESMF-compliant component for calculating emissions in atmospheric models. *Geoscientific Model Development* **7**, 1409–1417 (2014).

[66] Pickett-Heaps, C. A. *et al.* Magnitude and seasonality of wetland methane emissions from the Hudson Bay Lowlands (Canada). *Atmospheric Chemistry and Physics* **11**, 3773–3779 (2011).

[67] Wecht, K. J. *et al.* Spatially resolving methane emissions in California: constraints from the CalNex aircraft campaign and from present (GOSAT, TES) and future (TROPOMI, geostationary) satellite observations. *Atmospheric Chemistry and Physics* **14**, 8173–8184 (2014).

[68] Wecht, K. J., Jacob, D. J., Frankenberg, C., Jiang, Z. & Blake, D. R. Mapping of North American methane emissions with high spatial resolution by inversion of SCHIAMACHY satellite data. *Journal of Geophysical Research: Atmospheres* **119**, 7741–7756 (2014). 2014JD021551.

[69] Baker, D.F. *et al.* TransCom 3 inversion intercomparison: Impact of transport model errors on the interannual variability of regional CO_2 fluxes, 1988?2003. *Global Biogeochemical Cycles* **20**, GB1002 (2006).

[70] Michalak, A. M. & Kitanidis, P. K. A method for enforcing parameter nonnegativity in Bayesian inverse problems with an application to contaminant source identification. *Water Resources Research* **39** (2003). 1033.

[71] McLachlan, G. & Krishnan, T. *The EM Algorithm and Extensions.* Wiley Series in Probability and Statistics (Wiley, Hoboken, New Jersey, 2007).

[72] Miller, S. M., Michalak, A. M. & Levi, P. J. Atmospheric inverse modeling with known physical bounds: an example from trace gas emissions. *Geoscientific Model Development* **7**, 303–315 (2014).

[73] Kitanidis, P. *Introduction to geostatistics: applications to hydrogeology.* (Cambridge University Press, Cambridge, United Kingdom, 1997).

[74] Janssens-Maenhout, G. *et al.* EDGAR v4.3.2 global atlas of the three major greenhouse gas emissions for the period 1970–2012. *Earth System Science Data Discussions* **2017**, 1–55 (2017).

[75] Nisbet, E.G. *et al.* Rising atmospheric methane: 2007–2014 growth and isotopic shift. *Global Biogeochemical Cycles* **30**, 1356-1370 (2016).

[76] Schaefer, H. *et al.* A 21st-century shift from fossil-fuel to biogenic methane emissions indicated by $^{13}CH_4$. *Science* **352**, 80-84 (2016).

[77] Worden, J. *et al.* Reduced biomass burning emissions reconcile conflicting estimates of the post-2006 atmospheric methane budget. *Nature Communications* **8**, 2227 (2017).

[78] Schwietzke, S. *et al.* Upward revision of global fossil fuel methane emissions based on isotope database. *Nature* **538**, 88-91 (2016).

[79] Zazzeri, G. *et al.* Carbon isotopic signature of coal-derived methane emissions to the atmosphere: from coalification to alteration. *Atmospheric Chemistry and Physics* **16**, 13669-13680 (2016).

[80] Rigby, M. *et al.* Role of atmospheric oxidation in recent methane growth. *Proceedings of the National Academy of Sciences* **114**, 5373–5377 (2017).

[81] Turner, A. J., Frankenberg, C., Wennberg, P. O. & Jacob, D. J. Ambiguity in the causes for decadal trends in atmospheric methane and hydroxyl. *Proceedings of the National Academy of Sciences* **114**, 5367–5372 (2017).

[82] Montzka, S.A. *et al.* Small interannual variability of global atmospheric hydroxyl. *Science* **331**, 67–69 (2011).

[83] Hartmann, D. *et al.* *Climate Change 2013: The Physical Science Basis*, book section 2, 159–254 (Cambridge University Press, Cambridge, United Kingdom and New York, NY, USA, 2013).

[84] Naik, V. *et al.* Preindustrial to present-day changes in tropospheric hydroxyl radical and methane lifetime from the Atmospheric Chemistry and Climate Model Intercomparison Project (ACCMIP). *Atmospheric Chemistry and Physics* **13**, 5277–5298 (2013).
